# Endovascular Recanalization for Chronic Symptomatic Intracranial Vertebral Artery Total Occlusion

**DOI:** 10.1155/2014/949585

**Published:** 2014-09-03

**Authors:** Ziqi Xu, Ning Ma, Dapeng Mo, Edward Ho chung Wong, Feng Gao, Liqun Jiao, Zhongrong Miao

**Affiliations:** ^1^Department of Neurology, The First Affiliated Hospital of College of Medicine, Zhejiang University, No. 79 QingChun Road, Hangzhou, Zhejiang 310003, China; ^2^Department of Interventional Neuroradiology, Beijing Tiantan Hospital, Capital Medical University, No. 6 Tiantan Xili, Beijing 100050, China; ^3^Department of Medicine and Therapeutics, Chinese University of Hong Kong, Hong Kong; ^4^Department of Neurosurgery, Xuanwu Hospital, Capital Medical University, Beijing, China

## Abstract

*Purpose.* The outcome of recanalization in patients with chronic symptomatic intracranial vertebral artery (ICVA) total occlusion is poor. This paper reports the technical feasibility and long-term outcome of ICVA stenting in patients with chronic symptomatic total occlusion. *Methods.* Retrospective review of our prospectively maintained intracranial intervention database to identify patients with symptomatic total occlusion of ICVA with revascularization attempted >1 month after index ischemic event. *Results.* Eight patients (mean age 58 years) were identified. One had stroke and 7 had recurrent transient ischemic attacks. Four had bilateral ICVA total occlusion and 4 had unilateral ICVA total occlusion with severe stenosis contralaterally. Seven of 8 patients underwent endovascular recanalization, which was achieved in 6. Periprocedural complications included cerebellum hemorrhage, arterial dissection, perforation, and subacute in-stent thrombosis which occurred in 3 patients. One patient died of cerebellum hemorrhage. The other patients improved clinically after endovascular therapy. *Conclusions.* Stent-supported recanalization of ICVA total occlusion is technically feasible, and may become a viable treatment option in selected patients.

## 1. Introduction

Intracranial vertebral artery (ICVA) is a common site of atherosclerosis, often involved bilaterally [1, 2]. Patients with bilateral ICVA occlusion usually present with recurrent attacks of dizziness, visual disturbance, and ataxia, and some may be disabled by infarction of regions supplied by the posterior circulation [2]. While limited leptomeningeal collaterals may maintain baseline perfusion for most cases, they usually failed to provide sufficient blood flow during periods of increased oxygen demand, resulting in lifestyle-limiting symptoms. Development of endovascular intervention and improved operator experience have rendered angioplasty and stenting a potential treatment option for these patients but its safety and efficacy remains uncertain [3–5].

The incidence of the chronic ICVA occlusive disease in the Chinese patients may overweigh that of Caucasian patients [6]. To evaluate the feasibility and efficacy of the endovascular therapy for them is urgent. In this paper, we illustrated the feasibility of elective (at least 1 month after ischemic event) endovascular recanalization for symptomatic ICVA total occlusion and their long-term outcome.

## 2. Methods

### 2.1. Patient Selection and Data Collection

We retrospectively reviewed our prospectively maintained neurointerventional database from March 2007 to July 2012, to identify patients intended for ICVA recanalization. Inclusion criteria for analysis were (1) index ischemic stroke or transient ischemic attack in the posterior cerebral circulation of at least 1 month before; (2) unilateral ICVA total occlusion together with contralateral ICVA total occlusion, hypoplastic, absence, or severe stenosis (>70%) verified by computed tomography (CT), magnetic resonance (MR), or conventional angiography; (3) symptoms refractory to double antiplatelets (aspirin 300 mg/day and clopidogrel 75 mg/day) and strict risk factors management.

### 2.2. Endovascular Recanalization Protocol

**Figure 1:**
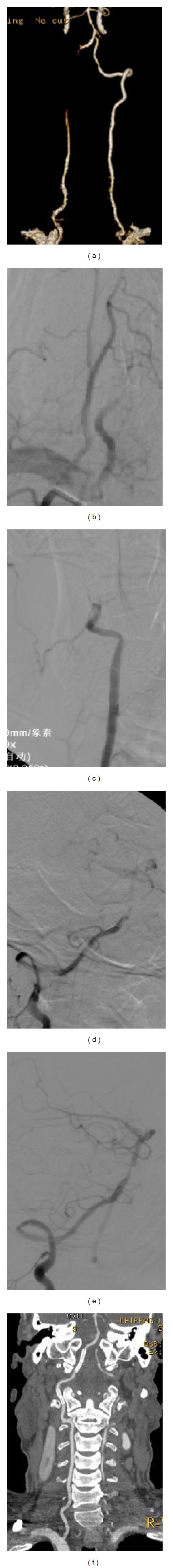
A 62-year-old man (case 3) with right intracranial vertebral artery (ICVA) total occlusion and tandem occlusion of the V3 by CTA (a) and DSA ((b), (c)). Contralateral severe stenosis of the V4 and tortuosity of the V1 were confirmed by CTA (a). The lesion was predilated with a 2 × 20 mm Invatec balloon and covered with a 3 × 18 mm Apollo stent for the V4 and a 3 × 15 mm Apollo stent for the V3 ((d), (e)). A 24-month CTA follow-up showed patency of the right V3 and V4 without in-stent stenosis (f).

**Figure 2:**
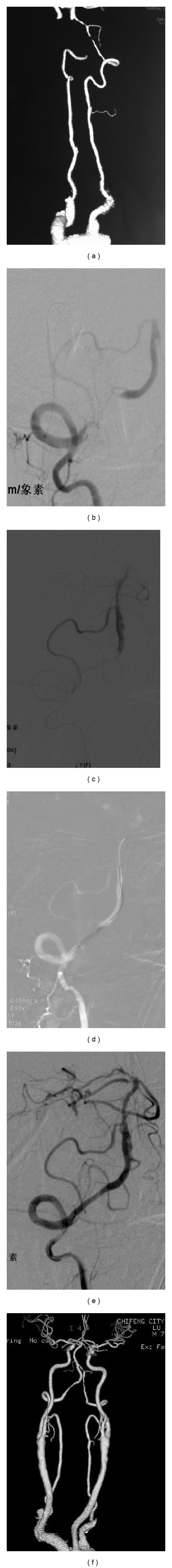
A 72-year-old man (case 6) with right intracranial vertebral artery (ICVA) total occlusion and contralateral severe stenosis confirmed by CTA (a) and DSA (b). The distal patency was confirmed by angiography through microcatheter (c). The lesion was predilated with a 2 × 20 mm Invatec balloon (d) and covered with a 3 × 19 mm Invatec balloon-expandable stent (e). A 24-month follow-up CTA showed mild in-stent stenosis (f).

Under general anesthesia, the course of the occluded ICVA was traversed and extrapolated with a coaxial assembly of Agility soft microguidewire (Cordis Corp) and Prowler 14 microcatheter (Cordis Corp). The microguidewire was withdrawn and distal patency beyond the occlusion confirmed by angiography through microcatheter. A long Transcend microguidewire (Stryker Neurovascular) was then exchanged. After withdrawing the microcatheter, the lesion was predilated with a balloon catheter and subsequently covered with a balloon-expandable stent or the Wingspan Stent System (Stryker Neurovascular) (Figures 1 and 2). The balloon and stent were sized based on measurement of the proximal ICVA as well as on the length of the occluded segment. Recanalization was defined as good antegrade perfusion of thrombolysis in cerebral ischemia (TICI) grade 3. Heparin anticoagulation therapy was maintained for the duration of the procedure. Blood pressure was tightly controlled postoperatively.

## 3. Results

Eight male patients (mean age, 58 years; range, 37–72 years) were analyzed in this study (Table 1). Of the 8 patients, 1 had acute stroke and 7 had recurrent transient ischemic attacks. Four had bilateral ICVA total occlusion and 4 unilateral ICVA total occlusion and contralateral severe stenosis. All patients had evidence of previous infarction in the vertebrobasilar distribution on MR imaging. Hyperlipidemia was present in all patients, 5 patients had hypertension, 4 patients had diabetes mellitus, and 5 patients had smoking history.

**Figure 3:**
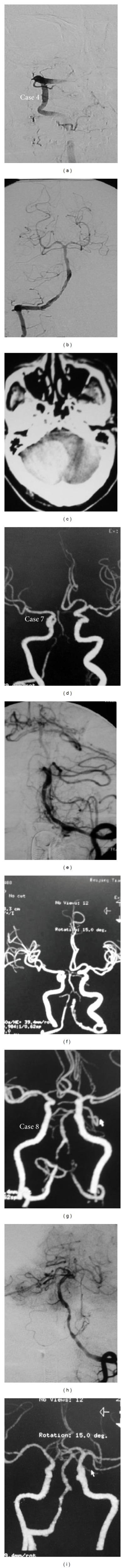
A 61-year-old man (case 4) with bilateral intracranial vertebral artery (ICVA) total occlusion (a). Following stenting of right ICVA (b), cerebellum hemorrhage due to perforation was found on the postprocedure CT (c). A 71-year-old man (case 7) with the left ICVA total occlusion and the right ICVA severe stenosis (d). D asymptomatic distal dissection and proximal perforation adjacent to the occlusion occurred during the procedure (e). The perforation resolved after balloon occlusion (f). A 37-year-old man (case 8) with the left ICVA total occlusion and the right ICVA severe stenosis (g). Both ICVA were recanalized but in-stent thrombosis occurred on the left ICVA as shown on day 1 after procedure CTA ((h), (i)).

Seven of 8 patients underwent endovascular recanalization eventually, as procedure was abandoned in one patient (case 5) after microcatheter angiography showed complete thrombosis in the distal lumen. Recanalization was achieved in 6 of 7 patients, as 1 patient (case 7) developed dissection. Periprocedural complications included cerebellum hemorrhage due to perforation in 1 patient (case 4) (Figures 3(a)–3(c)), asymptomatic distal dissection and proximal perforation adjacent to the occlusion in 1 patient (case 7) (Figures 3(d)–3(f)), and asymptomatic subacute in-stent thrombosis one day after the procedure in another (case 8) (Figures 3(g)–3(i)). One patient (case 4) died from cerebellar hemorrhage. Other patients were stable neurologically or improved after endovascular therapy. In the 6 recanalization patients, the 3 patients with follow-up angiography (Figures 1 and 2), 2 had mild in-stent stenosis (<50%). For the 3 patients without follow-up angiography, the lumen patency was confirmed with CT angiography in 2 patients and with transcranial Doppler in 1 patient.

## 4. Discussion

While modern diagnostic imaging techniques demonstrate ICVA and its lesions well, treatment options of patients with ICVA disease remain limited [1]. Our series showed that direct stenting without intra-arterial thrombolysis for symptomatic chronic ICVA occlusion is technically feasible. The technical success rate of 86% (6/7) was comparable to previously reported of 89% (8/9) in subacute to chronic basilar occlusion [7]. There are several important technical issues related to this challenging procedure. Firstly, great care must be taken in advancing the microguidewire tip, with gentle rotating movement to ensure its position within the true lumen. Secondly, after traversing the microcatheter through the occlusion, segmental angiograms while withdrawing the microcatheter from the tip of basilar artery to distal end of occlusion are crucial not only to evaluate the length of lesion, but also to look for signs of distal filling defects from thrombus. Thirdly, while the balloon-expandable stent system is more rigid than Wingspan system, it is preferred for patients with suitable smooth access path as delivery of the balloon-expandable stent does not require exchanging.

Periprocedural complications of our series demonstrated the double-edge sword effect of endovascular therapy for chronic ICVA occlusion. Perforation remains a real and devastating possibility, and temporary balloon occlusion may be a solution as used in case 7. Thromboembolism and perforator stroke are other potentially serious complications which did not occur in our series. One patient succumbed from procedural-related cerebellar hemorrhage but none experienced recurrent vertebrobasilar insufficiency symptoms during follow-up, although 2 of 3 patients developed mild in-stent restenosis confirmed with follow-up angiography. For this group of patients, provided periprocedural complications can be avoided, their long-term clinical outcome appeared promising. The small sample and uncontrolled design of this study weaken its relevance so a prospective randomized trial may be warranted to evaluate the safety and efficacy of such therapy in the future.

## 5. Conclusions

Stent-supported recanalization of chronic total occlusion of ICVA can be a viable option with an acceptable safety profile in selected patients.

## Figures and Tables

**Table 1:** Clinical summary of 9 patients undergoing endovascular recanalization of chronic intracranial vertebrobasilar occlusion.

No.	Age, yrs/sex	Symptoms on admission	Time between symptoms and treatment, mo	Image-documented vertebrobasilar occlusion, d	mRS score on admission	Success of recanalization	Occlusion site/balloon mm/stent mm	Periprocedural complication	Concomitant stenosis	Periprocedural complication	mRS Score at discharge	mRS score at latest follow-up	Clinical follow-up, mo	Angiography follow-up, mo	In-stent stenosis
1	52/M	Vertigo	5	8	2	Yes	LV4/ gateway 2 × 9/ Wingspan 3 × 20	None	RV4 total occlusion	None	1	0	63	60	Mild

2	59/M	Dizziness	5	14	2	Yes	LV4/ Invatec 2 × 15/ coroflex theca 3 × 13	None	RV4 total occlusion	None	1	0	47	NA	NA

3	62/M	Dizziness	6	5	1	Yes	RV4/ Invatec 2 × 20/ Apollo 3 × 18	None	RV3 total occlusion∗; LV4 severe stenosis	None	0	0	44	10	None

4	61/M	Vertigo and bilateral limb weakness	4	10	2	Yes	RV4/ gateway 3 × 20/ Wingspan 4 × 20; coroflex blue, 3.5 × 13; Wingspan 4.5 × 15	Cerebellum hemorrhage	LV4 total occlusion	Cerebellum hemorrhage	6	NA	NA	NA	NA

5	52/M	Dizziness and bilateral limb weakness	6	21	2	NA	RV4/none/none	NA	LV4 total occlusion	NA	2	NA	NA	NA	NA

6	72/M	Vertigo and bilateral limb weakness	6	40	2	Yes	RV4/ Invatec 2 × 20/ Invatec 3 × 19	None	LV4 severe stenosis^†^	None	1	0	29	24	Mild

7	71/M	Vertigo and loss of alance	3	27	2	Partial recanalization	LV4/ gateway 2 × 15/ Wingspan 3.5 × 20, Wingspan 3.5 × 20	Dissection and perforation	LV1 moderate stenosis; RC1 moderate stenosis; RV4 severe stenosis; LC1 severe stenosis^‡^	Dissection; arterial perforation	1	0	1	NA	NA

8	37/M	Blurred vision and vertigo	2	31	1	Yes	LV4/ gateway 2.25 × 15/ Wingspan 3 × 20	Subacute thrombosis	RV4 severe stenosis^§^	Subacute thrombosis	0	0	1	NA	NA

*RV3 treated with a 3 × 15 mm Apollo stent.

^†^LV4 treated with a 2.5 × 10 mm Invatec balloon and placement of a 2.5 × 8 mm Apollo stent.

^‡^LC1 treated with a 5 × 30 mm Viatrac balloon and placement of a 9 × 30 mm precise stent.

^§^RV4 treated with a 2.5 × 13 mm Apollo stent.
